# Health-related quality of life in different stages of chronic kidney disease and at initiation of dialysis treatment

**DOI:** 10.1186/1477-7525-10-71

**Published:** 2012-06-18

**Authors:** Agneta A Pagels, Birgitta Klang Söderkvist, Charlotte Medin, Britta Hylander, Susanne Heiwe

**Affiliations:** 1 Department of Medicine, Karolinska Institute, SE-17176, Stockholm, Sweden; 2 Department of Nephrology, Karolinska University Hospital, SE-17176, Stockholm, Sweden; 3 Department of Neurobiology, Care Sciences and Society, Karolinska Institute, SE-17176, Stockholm, Sweden; 4 Department of Clinical Sciences, Danderyd Hospital, SE-17176, Stockholm, Sweden

**Keywords:** Quality of life, Health status, Chronic kidney disease, Cross-sectional studies, Comorbidity

## Abstract

**Objectives:**

To evaluate health-related quality of life (HRQoL) in patients in different stages of chronic kidney disease (CKD) up to initiation of dialysis treatment and to explore possible correlating and influencing factors.

**Methods:**

Cross-sectional design with 535 patients in CKD stages 2–5 and 55 controls assessed for HRQoL through SF-36 together with biomarkers.

**Results:**

All HRQoL dimensions deteriorated significantly with CKD stages with the lowest scores in CKD 5. The largest differences between the patient groups were seen in ‘physical functioning’, ‘role physical’, ‘general health’ and in physical summary scores (PCS). The smallest disparities were seen in mental health and pain. Patients in CKD stages 2–3 showed significantly decreased HRQoL compared to matched controls, with differences of large magnitude - effect size (ES) ≥ .80 - in ‘general health’ and PCS. Patients in CDK 4 demonstrated deteriorated scores with a large magnitude in ‘physical function’, ‘general health’ and PCS compared to the patients in CKD 2–3. Patients in CKD 5 demonstrated deteriorated scores with a medium sized magnitude (ES 0.5 – 0.79) in ‘role emotional’ and mental summary scores compared to the patients in CKD 4. Glomerular filtration rate <45 ml/min/1.73 m², age ≥ 61 years, cardiovascular disease (CVD), diabetes, C-reactive protein (CRP) ≥5 mg/L, haemoglobin ≤110 g/L, p-albumin ≤ 35 g/L and overweight were associated with impaired HRQoL. CRP and CVD were the most important predictors of impaired HRQoL, followed by reduced GFR and diabetes.

**Conclusions:**

Having CKD implies impaired HRQoL, also in earlier stages of the disease. At the time for dialysis initiation HRQoL is substantially deteriorated. Co-existing conditions, such as inflammation and cardiovascular disease seem to be powerful predictors of impaired HRQoL in patients with CKD. Within routine renal care, strategies to improve function and well-being considering the management of co-existing conditions like inflammation and CVD need to be developed.

## Background

When evaluating and improving health care in chronic diseases, symptoms, function in daily life and well-being are important patient outcomes [1]. Health-related quality of life (HRQoL) is a significant key indicator of how a condition affects the patient’s life. HRQoL assessments can therefore identify possible problem areas related to health experiences. The concept of HRQoL builds on WHO’s definition of health [2] and has been defined as the subjective assessment of the impact of disease and its treatment across the physical, psychological and social domains of functioning and well-being [3]. It is characterized by being multidimensional (reflecting at minimum physiological, psychological and sociological aspects), temporal and subjective [2].

Chronic kidney disease (CKD) is defined as the presence of kidney damage or a glomerular filtration rate (GFR) < 60 ml/min/1.73 m² for ≥ 3 months [4], with a prevalence of approximately 10% of the adult population [5]. Stages of CKD and levels of renal function are described in Table 1, the higher the CKD stage, the more severe the renal insufficiency. Most CKD tend to progress, and with declined kidney function multiple disorders gradually develop, such as anemia, hypertension, inflammation (i.e. chronic activation of the immune system), malnutrition metabolic and mineral-bone disorders [6-8]. CKD 1–3 are not usually considered to impact on the individual’s health experience, although some disturbances may already have emerged. However, in CKD 4 the individual perceives an increasing amount of symptoms which may affect the HRQoL [7]. Fatigue, muscle weakness, restless legs, cramps, itching, nausea and loss of appetite [9-11] are frequently reported symptoms. Conditions like malnutrition, anemia, cognitive dysfunction, sleep disorders, depression, reduced social interaction, physical and sexual functioning and co-morbidities like diabetes and cardiovascular disease (CVD) also impair HRQoL in CKD patients [12-14]. Impaired HRQoL is well described among patients on dialysis treatment [14,15]. Low HRQoL scores in dialysis patients are further strong and independent predictors of hospitalization and mortality [16-18]. However, some studies have demonstrated deteriorated HRQoL also in early stages of CKD, especially in physical health [19-21] but also in mental health [22]. When following patients in CKD 3–5 up to four years, it was shown that HRQoL deteriorated over time, especially in those with a history of congestive heart failure [20]. Impaired HRQoL have also been shown shortly before (0–4 weeks) initiation of dialysis treatment [23].

**Table 1:** Stages of chronic kidney disease (CKD) related to levels of kidney function, i.e., glomerular filtration rate (GFR) (National Kidney Foundation, 2002)

**CKD stage**	**Description**	**GFR (ml/min/1.73 m^2^)**
1	Kidney damage with normal or increased kidney function	≥ 90
2	Kidney damage with mildly diminished kidney function	60 – 89
3	Moderately reduced kidney function	30 – 59
4	Severely decreased kidney function	15 – 29
5	Kidney failure	< 15

Few studies have examined HRQoL patterns in different stages of CKD which indicate that more knowledge is needed. The objective of this study was therefore to evaluate HRQoL in patients with different stages of CKD up to initiation of dialysis treatment and to explore possible correlating and influencing factors. It was assumed that HRQoL would decline progressively with impaired renal function but also that co-morbidity, age, gender, inflammation, anemia, hypertension and altered nutritional markers would impact negatively on HRQoL.

## Materials and methods

### Patients and study design

In this cross-sectional study 535 patients in CKD 2–5, with a GFR ranging from 69 to 2 ml/min/1.73 m², and 55 controls from the Stockholm region in Sweden were assessed for HRQoL through the SF-36 questionnaire (see flow chart shown in Additional file 1: Table S1). Register data from two prospective observational studies (PROGRESS and PAUS) and the local Swedish Renal Registry (SRR) were collected and merged according to Additional file 1: Table S1. The participants in the PROGRESS (‘*Factors impacting progress of renal insufficiency*’) cohort (n = 104) were recruited by convenience at a renal outpatient clinic during 2002–2009. These participants had a renal function corresponding to CKD 2–3 or 4–5. In the PAUS (‘*Prospective study of renal replacement therapy in Stockholm*’) cohort, 532 patients were recruited consecutively from eight nephrology units when initiating dialysis (SF-36 was collected at or up to two weeks after first dialysis session) treatment during 2000–2005. Of these, 330 patients from seven units participated in the HRQoL survey. In this cohort, 97% of the participants had a renal function, corresponding to CKD 5. From the local SRR cohort (n = 468), 116 patients were recruited by convenience for HRQoL assessments in connection to visits at a renal outpatient clinic during 2004–2009. These participants had a renal function corresponding to CKD 3–5. Controls, matched for age, sex and living area to the CKD 2–3 patients were recruited. Of these, 31 were randomly selected from the Swedish Register of the Total Population and 24 recruited through the web site of the regional university hospital. Inclusion criteria for the controls were GFR ≥ 80 ml/min/1.73 m², absence of kidney disease, cardiovascular disease (CVD), diabetes and any ongoing medication. All participants had given their informed consent.

### Questionnaire and procedure

#### SF-36 questionnaire

The 36-item short-form questionnaire (SF-36) [24] is a self-administered general HRQoL questionnaire, not specific to any disease or treatment group. The SF-36 is covered by a conceptual model of HRQoL [25] and includes 36 items that yield an 8- dimension profile on a 100-point scale, a higher score indicates a better perceived health state. The eight dimensions are: Physical function (PF), Role limitations caused by physical problems (RP), Pain (BP), General health (GH), Vitality/energy (VT), Social function (SF), Mental health/emotional well-being (MH) and Role limitations caused by emotional problems/mental health (RE). The items refer to perceived health status during the last 4 weeks. The PF, RP, BP and GH dimensions are usually summarized into a physical composite summary (PCS) and the VT, SF, MH and RE dimensions summarize to a mental composite summary (MCS) [24,26]. The mean scores of the Swedish reference population (n = 8930) aged 15–93 are: PF = 87.9, RP = 83.2, BP = 74.8, GH = 75.8, VT = 68.8, SF = 88.6, RE = 85.7, MH = 80.9, the mean summary scores (n = 8004) are: PCS = 50 and MCS = 50 [27]. The SF-36 (version 1.0) was used as it covers relevant domains of function and well-being, and most of the items in SF-36 are considered to have good sensitivity and responsiveness [28]. SF-36 has been used world-wide and been recognized in various contexts and thus permits comparison within and between other conditions. It is valid and reliable [27,29] and recommended by the National Kidney Foundation guidelines [7].

#### Procedures and clinical measures

The patients from all three cohorts and the controls were asked to complete the SF-36 by self-administration in connection with their visits at the nephrology units. Biomarkers for all participants (including the controls) were blood test analyses, blood pressure, weight and Body Mass Index (BMI). Blood test analyses were haemoglobin (Hb), albumin, C-reactive protein (CRP), phosphate, parathyroid hormone (PTH) and creatinine. Moreover, GFR was determined in all participants. In the controls and the patients in CKD 2–3 GFR was examined by Iohexol-clearance, and in all other patients GFR was estimated using the Modification of Diet in Renal Disease (MDRD) formula [30,31]. Blood pressure was measured in horizontal or sitting position and mean arterial pressure (MAP) was calculated. Weight measures were performed and BMI was calculated.

## Data analysis/statistical methods

IBM® SPSS® Statistics software (SPSS Inc., Chicago, IL, USA 2006), version 15 and 20 were used for the statistical analyses. Raw scores from the questionnaires were transformed to scale scores using the software syntax for SF-36 [27]. Data was analyzed and presented according to outcome of normal distribution tests. Chi-square test was used to analyze differences in nominal-level variables. Independent *t*-test was used to compare mean HRQoL scores related to categorized correlates (gender, history of co-morbidity, age, GFR, Hb, albumin, CRP, blood pressure, BMI). Cut-off value for age was set at the mean for the whole patient group, i.e. 61 years. Other correlate cut-off values were set in accordance to clinical guidelines, research findings and expertise knowledge, such as GFR at 45 ml/min/1.73 m², Hb-value at 110 g/L, CRP at 5 mg/L, albumin at 35 g/L, MAP at 110 mmHg and BMI at 20 and 30. Data from CKD 2 and 3 were pooled, as there were no significant differences in SF-36 scores between these groups. Differences in HRQoL between CKD 2–3 and the matched controls were evaluated by the Mann- Whitney *U*-test. HRQoL differences between the three patient groups were analysed by the ANOVA one-way test. The magnitude of differences in HRQoL scores was assessed using Cohen’s *d* formula [32]. Cohen’s *d* is a standardized measure of effect size (ES) and is computed as the difference between the mean scores of the compared groups divided by the pooled within-group standard deviation (SD). The Cohen’s *d* was computed, using the calculator elaborated by Becker [33]. According to Cohen [32], benchmarks for evaluating the importance of differences are: ES values < 0.49 are considered as small, values of 0.50 – 0.79 as medium, and values ≥ 0.80 are considered as large. Predicting factors were computed through multiple linear regression analyses, using the enter method. A random sample of 70 patients within CKD 5 (n = 394) was extracted using a random number table.

The study was approved by the Regional Ethical Review Board in Stockholm, Sweden.

## Results

Demographic and biomarker characteristics are presented in Table 2, with the patients divided into three groups according to their renal function; CDK 2–3, with GFR range 69 – 31 ml/min/1.73 m² (n = 54), CKD 4, with GFR range 29 – 15 ml/min/1.73 m² (n = 87) and CKD 5, with GFR range 14 – 2 ml/min/1.73 m² (n = 394) All biomarkers except BMI deteriorated significantly (p = .000) across the CKD stages. CRP levels of ≥10 mg/L were significantly (p = .04) more frequent among the men (40%) than the women (31%). CVD was significantly more frequent in CKD 4–5 (53%) than in CKD 2–3 (13%) (p = .000), and were also more frequent among the men (56%) than the women (36%) (p = .000).

**Table 2:** Participants, grouping according to Chronic Kidney Disease (CKD) stages, renal diagnoses, co-morbidity, demographic and laboratory data

	**All patients (n = 535)**	CKD stage 2 – 3* GFR range 31–69 (n = 54)	CKD stage 4** GFR range 15–29 (n = 87)	CKD stage 5** GFR range 2–14 (n = 394)	Controls* (n = 55)
Age, M (SD)	61 (15)	47 (11.2)	62 (15.7)	62 (14.4)	48 (10.6)
Females, n (%)	175 (33)	22 (41)	35 (40)	118 (30)	22 (40)
BMI, Md (IQR)	25 (22–28)	25 (22–28)	25 (23–28)	25 (22–28)	24 (22–27)
MAP, M (SD)	101 (15.4)	96 (12.8)	94 (13.4)	103 (5.6)	88 (10.4)
GFR, ml/min/1.73 m²§, M (SD)	15 (16)	60 (6.6)	19 (3.1)	8 (3)	99 (12)
Hb, g/L, M (SD)	115 (17)	136 (14.2)	122 (12.6)	111 (15.6)	142 (11.5)
p-Albumin,g/L, M (SD)	34 (5.5)	38 (3.6)	36 (4.4)	33 (5.6)	40 (2.8)
p-Phosphate, mmol/L, M (SD)	1.8 (0.6)	1.1 (0.2)	1.4 (0.3)	2.0 (0.6)	1.1 (0.2)
PTH, ng/L, Md (IQR)	160 (76–303)	46 (35–56)	146 (91–204)	201 (103–345)	43 (38–53)
CRP, mg/L, Md (IQR)	5 (4–18)	2.4 (1–4.4)	4 (1.6–8.9)	6 (5–23)	0.89 (0.5–2.3)
CRP = 5–10 mg/L, n (%)	182 (34)	5 (9)	17 (20)	160 (41)	2 (4)
CRP = >10 mg/L, n (%)	163 (30)	4 (7)	13 (15)	146 (37)	3 (5)
n, (%)					
Renal vascular disease	115 (22)	1 (2)	22 (25)	92 (23)	
Primary glomerulonephritis	95 (18)	18 (33)	12 (13)	65 (17)	
Familial, hereditary renal disease	58 (11)	12 (20)	6 (7)	40 (10)	
Secondary glomerular systemic diseases^a^	46 (9)	2 (4)	5 (6)	39 (10)	
Other renal diagnose	101 (19)	13 (24)	20 (23)	68 (17)	
CVD history ^b^	264 (49)	7 (13)	49 (56)	208 (53)	
Diabetes history ^c^	158 (30)	11 (20)	40 (46)	107 (27)	
Other co-morbidity^d^	119 (22)	2 (4)	22 (25)	95 (24)	

* *GFR* = glomerular filtration rate in ml/min/1.73 m², examined by Iohexol-clearance.

** GFR estimated by MDRD. BMI = Body Mass Index, MAP = Mean Arterial Pressure, Hb = Hemoglobin. ^a^ Secondary glomerular systemic diseases except for diabetic nephropathy. ^b^ Cardiovascular disease (CVD) includes cardiac infarction, congestive heart failure, stroke/TIA, atherosclerosis/peripheral vascular disease. ^c^ Diabetic nephropathy or diabetes as co-morbidity. ^d^Other co-morbidity includes malignancy, chronic respiratory disease, chronic liver disease and rheumatic disease.

**Figure 1:**
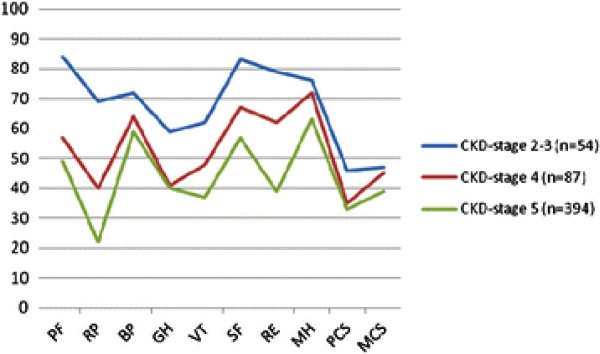
**HRQoL domains and summary scores (M) in different stages of Chronic Kidney Disease (CKD).** PF = Physical functioning, RP = Role physical, BP = Bodily pain, GH = General health, VT = Vitality, SF = Social functioning, RE = Role emotional, MH = Mental health, PCS = Physical summary scores, MCS = Mental summary scores.

**Figure 2:**
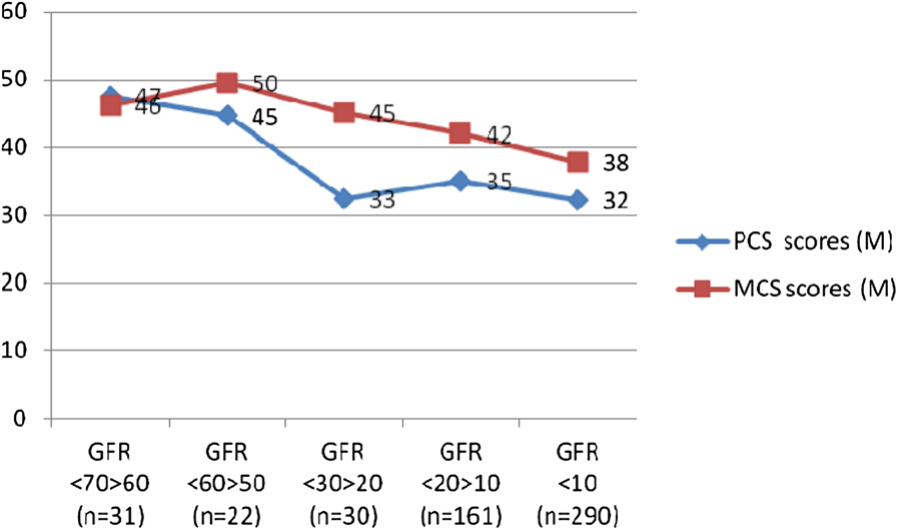
**Mean Physical Composite summary (PCS) and Mental Composite Summay (MCS) scores related to declining levels of kidney function/Glomerular Filtration Rate (GFR), ml/min/1.73 m².** GFR span <50 > 30 not shown, since it was covered by only one patient.

As shown in Table 3 and in Figures 1 and 2, the HRQoL scores in all dimensions impaired progressively and significantly (p = .000) across renal function levels and CKD stages. The lowest scores were found in CKD 5. The largest differences between CKD stages were seen in ‘physical function’, ‘role physical’, ‘general health’ and PCS. The smallest disparities were seen in ‘pain’ and ‘mental health’. These smaller differences still met the criteria for minimal clinically important difference (MCID) of 3–5 score units [34]. Score differences found between CKD 4 versus 5 in ‘general health’ and PCS did not approach MCID. This was also observed in ‘mental health’ and MCS between CKD 2–3 versus 4.

**Table 3:** HRQoL dimensions in different stages of Chronic Kidney Disease (CKD) and in healthy controls

**HRQoL- dimensions**	**Controls vs CKD 2–3#**	**Controls (n = 55) Md (IQR)CI**	CKD 2–3* (n = 54) GFR range 31–69 Mean (SD),CI	CKD 4* (n = 87) GFR range 15–29 Mean (SD),CI	CKD 5* (n = 394) GFR range 2–14 Mean (SD),CI	**CKD 2–3 vs CKD 4–5##**
Physical	Z = −3.53	100	83.7 (22)	57 (28.5)	49.2 (28.7)	F = 67.1
functioning	p = .000	(95–100)	77.7–89.7	50.9–63	46.3–52	p = .000
(PF)		94.2–99				
Role	Z = −3.08	100	69 (37.3)	39.9 (45)	22.1 (35.6)	F = 64.3
physical	p = .002	(100–100)	58.8–79.2	30.4–49.5	15.5–25.6	p = .000
(RP)		78.5–95.1				
Bodily	Z = −2.51	84	71.5 (27.2)	63.8 (30.3)	58.8 (31.4)	F = 7.1
Pain	p = .012	(74–100)	64.1–79	57.3–70.2	55.7–61.9	p = .008
(BP)		80.9–89.4				
General	Z = −5.63	85	59.4 (21.5)	40.8 (17.8)	40.2 (18.9)	F = 49.3
health	p = .000	(77–95)	53.5–65.3	37–44.6	38.3–42.1	p = .000
(GH)		78.9–86.8				
Vitality,	Z = −2.29	75	61.5 (25.1)	48.3 (26.1)	36.5 (23.9)	F = 41.3
energy	p = .022	(65–85)	54.6–68.3	42.8–53.9	34.2–38.9	p = .000
(VT)		68.2–77.8				
Social	Z = −2.39	100	83.3 (22.5)	67 (28.4)	57 (30.7)	F = 33
functioning	p = .017	(88–100)	77.2–89.5	60.9–73	53.9–60	p = .000
(SF)		90.3–96.9				
Role	Z = −2.59	100	79 (35.6)	62.1 (43.5)	38.8 (42.5)	F = 34.3
emotional	p = .009	(100–100)	69.3–88.7	52.8–71.3	34.6–43	p = .000
(RE)		86.6–98.9				
Mental	Z = −2.39	84	75.9 (18.2)	71.6 (18.7)	62.9 (23.1)	F = 13
health	p = .017	(80–92)	71–80.9	67.7–75.6	60.6–65.1	p = .000
(MH)		81.8–87.6				
Physical	Z = −4.35	55	45.8 (10.4)	34.9 (11.9)	32.9 (10.5)	F = 66.6
Composite	p = .000	(52.4–57.3)	43–48.6	32.4–37.4	31.9–33.9	p = .000
summary		51.4–55.1				
(PCS)						
Mental	Z = −1.92	53	47.4 (10.6)	45 (12.4)	38.5 (12.8)	F = 17.9
composite	p = .055	(48.6–56)	44.5–50.3	42.4–47.6	37.2–39.7	p = .000
summary		49.3–53.5				
(MCS)						

* CKD-stage according to Table 1**. GFR = glomerular filtration rate in ml/min/1.73 m².**

# Post hoc Mann–Whitney *U*-Test. ## ANOVA One-way Test df = 533.

Confidence interval at 95% interval

**Figure 3:**
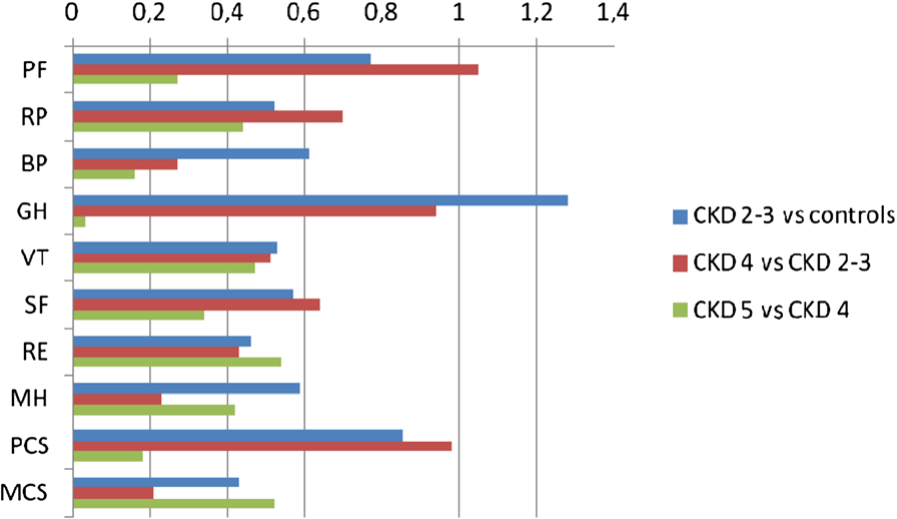
**Effect Sizes in HRQoL domains and summary scores (M) in different stages of Chronic Kidney Disease (CKD) and in controls.** PF = Physical functioning, RP = Role physical, BP = Bodily pain, GH = General health, VT = Vitality, SF = Social functioning, RE = Role emotional, MH = Mental health, PCS = Physical summary scores, MCS = Mental summary scores.

The patients in CKD 2–3 (GFR range 69–31 ml/min/1.73 m²) had significantly lower scores on all HRQoL dimensions than the matched controls (Table 3). ‘General health’ and PCS reached an ES of large magnitude (1.28 and 0.85 respectively) between CKD 2–3 and controls (Figure 3). ‘Role emotional’ and MCS both had ES of small magnitude (<0.49). All other dimensions had a medium sized ES (0.50–0.79).

The patients in CDK 4 demonstrated deteriorated scores with a large magnitude in ‘physical function’ (ES = 1.05), ‘general health’ (ES = 0.94) and PCS (ES = 0.98) compared to those in CKD 2–3 (Figure 3). ‘Role physical’ (ES = 0.70), ‘vitality’ (ES = 0.51) and ‘social functioning’ (ES = 0.64) showed differences of medium magnitude.

The patients in CKD 5 demonstrated deteriorated scores with a medium sized magnitude in ‘role emotional’ (ES = 0.54) and MCS (ES = 0.52) compared to those in CKD 4 (Figure 3). All other differences between these groups had small ES.

### Categorized correlates and HRQoL

Categorized correlates (GFR, age, gender, history of CVD, diabetes, Hb, albumin, CRP, MAP, BMI) and PCS and MCS scores are shown in Table 4. All significant differences (p < .05) met the required MCID for SF-36. Patients with GFR < 45 ml/min/1.73 m² as well as CRP ≥ 5 mg/L, Hb ≤ 110 g/L and albumin ≤35 g/L had significantly lower scores on all HRQoL dimensions. Among those with CVD, the PCS and all physical subscales were significantly lower than in those with no history of CVD (p < .05). In those with diabetes, PCS and ‘vitality’ were significantly lower compared to those without diabetes. Patients with overweight (BMI > 30) had significantly lower scores on PCS, ‘physical function’ (p = .006) and pain (p = .041) than their counterparts. However, no difference was seen between patients with BMI ≤ 20 compared to those with BMI >20. The group aged ≥61 years showed lower scores on PCS, ‘physical function’ and ‘role physical’ than the younger group, all at a significant level (p = .000). MCS was not affected significantly by age, although the ‘role emotional’ subscale was impaired among those ≥61 years than in the younger group (p = .004). Gender did not affect HRQoL significantly, nor did hypertension (MAP > 110 mmHg).

**Table 4:** Categorized correlates and HRQoL summary scores

			** PCS**		** MCS**	
**Parameter**		**N**	**Mean (SD)**	**p-value**	**Mean (SD)**	**p-value**
GFR	<45	482	33.2 (10.7)	.000	39.7 (13)	.000
	≥45	53	46.3 (10)		47.6 (10.6)	
Age	<61	281	37.6 (11.2)	.000	39.8 (13.4)	ns
	≥61	254	31.1 (10.5)		41.1 (12.5)	
Gender	Male	360	34.6 (11.5)	ns	40.4 (13)	ns
	Female	175	34.4 (11.1)		40.5 (12.8)	
CVD	Yes	264	29.6 (9.9)	.000	39.4 (12.7)	ns
	No	271	39.4 (10.5)		41.4 (13.2)	
Diabetes	Yes	158	30.4 (10.8)	.000	40.1 (13.1)	ns
	No	377	36.2 (11.2)		41.2 (12.6)	
Hb	≤110 g/L	205	31.0 (10.2)	.000	36.7 (12.9)	.000
	>110 g/L	330	36.7 (11.5)		42.8 (12.5)	
p-Albumin	≤35 g/L	306	32.0 (10.8)	.000	38.1 (12.9)	.000
	>35 g/L	227	37.9 (11.3)		43.6 (12.4)	
CRP	<5 mg/L	132	41.7 (11.2)	.000	43.6 (12.4)	.000
	≥5 mg/L	345	31.9 (10.5)		38.7 (13.1)	
MAP	≤110 mmHg	403	34.1 (11.7)	ns	40.7 (12.9)	ns
	>110 mmHg	125	36.1 (10.1)		39.9 (12.8)	
BMI	≤30	439	35.3 (11.4)	.003	40.7 (12.8)	ns
	>30	76	31.2 (10.8)		41.1 (13.2)	
BMI	≤20	55	36.6 (11.7)	ns	40.6 (14.2)	ns
	>20	460	34.4 (11.3)		40.8 (12.7)	

PCS = Physical Composite Summary, MCS = Mental Composite Summary, GFR = glomerular filtration rate in ml/min/1.73 m², CVD = Cardiovascular disease, Hb = Hemoglobin CRP = C-Reactive Protein, MAP = Mean Arterial Pressure, BMI = Body Mass Index.

Significance level: p < .05 ns = not significant.

### Multiple regression analyses

Multiple linear regression analyses were performed with the response variable ‘PCS’ and ‘MCS’ respectively. Ten explanatory variables were included: GFR, age, gender, CVD, diabetes, Hb, log CRP, p-albumin, MAP and BMI. When checking the assumptions (sample size, outliers, multicolinearity, singularity, normality, linearity and homoscedasticity), GFR was not normally distributed as the majority of the patients belonged to CKD 5. A random sample of 70 patients (with similar demographic distribution) was therefore drawn from the CKD 5 group and collapsed with CKD 2–4 data, creating a CKD 2–5 (n = 211) group.

A significant model for PCS emerged (F_5,170_ =31.062, p = .000). The regression was a rather good fit; which means that 46,2% of the variance in PCS was explained by the model (Adjusted R square = .462). Five of the ten explanatory variables had a significant predictive capacity. Out of the model’s explanatory variables, ‘CRP’ (Beta = −.279, p = .000) and ‘CVD’ (Beta = −.233, p = 002) showed the highest levels of explanation, followed by ‘GFR’ (Beta = .191, p = .003), ‘diabetes’ (Beta = −.160, p = .008) and ‘age’ (Beta = −.158, p = 021). The variables gender, p-albumin, Hb, MAP and BMI showed no significant predictive capacity. No significant model emerged with MCS as response variable. In the final model for MCS only one of the explanatory variables demonstrated a significant predictive capacity: ‘CRP’ (Beta = −.271, p = .001). The regression was a poor fit; only 11.7% of the variance in MCS was explained by the model (Adjusted R square = .117, F_5,170_ =5.626, p = .000). In summary, the results of the multiple regression analyses showed that CRP and CVD in the individual were the most prominent predictors for impaired PCS among adults with CKD 2–5, followed by GFR, diabetes and age.

## Discussion

Some limitations of the present evaluation ought to be considered. The patients were not randomly selected and neither were all of the controls, which could increase the risk for bias. Moreover, the group sizes in different CKD stages and GFR levels were disproportionate, with most participants in CKD 5. This, as well as the context for this study setting, has to be considered when interpreting the results. However, the multiregression analyses were performed with more proportional group sizes. Unfortunately, the study did not cover patients in GFR levels between 31–50 ml/min/1.73 m², but indicated that this GFR span may embed a turning point for a pronounced drop in PCS (Figure 2). Thus, our results confirm previous findings that GFR values around 45 ml/min/1.73 m² seem to be a dividing line for drop in HRQoL, especially in PCS [19]. Gender distribution was somewhat skewed with a majority of males, but this is in accordance with the gender distribution in CKD. Furthermore, a cross-sectional design is limited by only providing a snapshot and its difficulties to make causal inference. Thus the evolution of the participants’ HRQoL was not followed over time in this study, which may be of importance when interpreting the illness trajectory. Follow-up studies on HRQoL are still rare. Therefore, this should be focused on in future research. When interpreting a HRQoL instrument, one has to consider its limitations. Ceiling and floor effects may skew the results. The individuals’ assessments of their health status are strongly subjective and affected by surrounding factors like cultural aspects and environmental changes, which should be taken into account when interpreting and comparing results of HRQoL [35]. Furthermore, the response shift phenomenon, i.e. the patients’ adaption to or recalibration of their health condition may have influenced the individual responses in this study [36]. One can also presume that the patient’s awareness of the diagnosis or ‘labeling’ phenomenon may influence the individual’s health perception in asymptomatic conditions [13]. In this study solely biological variables were collected for correlation analyses. This has to be kept in mind when drawing conclusions. If non-biological variables (such as psycho-social aspects, illness representations, sense of coherence, locus of control, self-efficacy, coping strategies and self-management) had been assessed, this might have contributed to a more holistic perspective of components correlating to and predicting HRQoL – especially the mental domains - in this patient group. Moreover, one has to consider that the SF-36 does not capture all dimensions that may be included in HRQoL for patients with CKD, such as for example sleep, sexual and cognitive functioning. Strengths of this study are the large number of patients, especially in CKD 5, which has not been well studied previously regarding HRQoL. This study provides insights into the changes in HRQoL throughout the CKD illness trajectory, and contributes to providing more knowledge regarding HRQoL also in early CKD stages. The study also highlights the relationship between HRQoL and inflammation and CVD in this patient group.

In summary, the results showed that all HRQoL dimensions deteriorated significantly across CKD stages, with the lowest scores in CKD 5. The largest differences between the patient groups were seen in ‘physical functioning’, ‘role physical’, ‘general health’ and in PCS. The smallest disparities were seen in ‘mental health’ and ‘pain’. Patients in CKD stages 2–3 showed significantly decreased HRQoL compared to matched controls, with differences of large magnitude in ‘general health’ and PCS. Patients in CDK 4 demonstrated deteriorated scores with a large magnitude in ‘physical function’, ‘general health’ and PCS compared to those in CKD 2–3. Patients in CKD 5 showed deteriorated scores with a medium sized magnitude in ‘role emotional’ and MCS compared to those in CKD 4. GFR < 45 ml/min/1.73 m², age ≥ 61 years, CVD, diabetes, CRP ≥ 5 mg/L, Hb ≤ 110 g/L, p-albumin ≤ 35 g/L and overweight were all associated to impaired HRQoL, indicating that HRQoL related to renal function level, as well as to other conditions associated to CKD, like inflammation and CVD. CRP and CVD emerged as the most important predictors of impaired HRQoL, followed by reduced GFR and diabetes.

Present results indicate that both PCS and MCS were significantly impaired across CKD stages. As expected, the lowest HRQoL scores were seen in the patients with the most declined renal function, substantially deviating from the Swedish reference population [27]. The decline in HRQoL with deteriorating renal function is congruent with previous findings [20]. The mean scores of the patients in CKD 5 in this study (of which 80% were initiating dialysis treatment) were lower than among those in a Dutch study that assessed HRQoL 0–4 weeks prior to dialysis initiation [23]. This confirms the continuous deterioration of HRQoL with more advanced disease stages. Compared with previous HRQoL assessments, the current patients in CKD 5 showed even worse scores than in several dialysis populations [37], suggesting that this period of the disease trajectory may be exceptionally vulnerable. The time period right before and at initiating dialysis treatment can be described as a transitional state, where the situation often appears as fragile and uncertain to the patient. In addition to decline in physical health, stress, anxiety and depression often occur in the period preceding dialysis initiation [38-40]. It is therefore reasonable that also the mental dimensions should be reflected in HRQoL assessed at this point.

The results show that CKD has a negative impact on HRQoL – especially in the physical domains - already in earlier stages of the disease. These results are in line with findings in previous studies [19,20,22]. The patients in CKD 2–3 (with GFR range 69–31 ml/min/1.73 m².) scored significantly lower than the controls in all HRQoL dimensions, with the largest differences shown in ‘general health’ and PCS. The scores were also lower compared to the Swedish reference population [27]. This indicates that CKD even at an early stage seems to imply restrictions in daily life, though it has been considered not to impact the individual’s health experience. Little is still known regarding illness perceptions and health experiences in this patient group, why more research is necessary on this topic.

The associations between GFR, CVD, diabetes, decreased Hb- and p-albumin levels and impaired HRQoL might be expected and are congruent with findings in other studies [19,20]. However, the GFR level did not – as assumed - show any prominent predictive capacity in the multiple regression analyses. Interestingly, this study instead demonstrates that inflammation and CVD seem to be powerful predictors of impaired HRQoL in patients with CKD. Moreover, the results indicate that a relatively moderate increase in CRP may affect HRQoL. These findings highlight that key elements concerning HRQoL in CKD patients are still not settled and indicate that more attention should be paid to partners like inflammation and CVD. To our knowledge, the relationship between inflammation and HRQoL in this patient group is still not very well documented. It has previously been shown that increased levels of inflammation related cytokines were associated with deteriorated self-rated health [41]. Our results showed elevated CRP levels across the CKD stages, with increased occurrence of CRP > 5 mg/L with declined kidney function (Table 2). This is in line with previous findings [42,43] and the presumption of a chronic low-grade inflammation process, starting already in early stages of CKD [44]. Besides being an inflammation marker, CRP has also been pointed out as a strong predictor of CVD events [45,46]. Moreover, and congruent with previous research [47,48], about half of the patients had a history of CVD, and about a third had diabetes (Table 2). Conditions like inflammation, CVD and diabetes often appear in CKD patients, share risk factors and affect the HRQoL. By screening for them at an early stage the possibility for treatment and secondary prevention of these factors increases and thereby also contributes to improved well-being and function.

Research within renal care [19,20], in other conditions like chronic heart failure [49] and also in the Swedish reference population [27] have demonstrated women reporting worse HRQoL than the men. However, no association was found between HRQoL and gender in current results. This finding was identified even though a history of CVD and higher CRP-levels were more common among males than in females.

The physical HRQoL domains showed large impairments across the CKD stages in this study. The patients in CKD 4 had impaired scores in ‘physical function’ , ‘general health’ and PCS compared to the patients in CKD 2–3. The decline in ‘vitality’ indicates that the deteriorated physical function might be connected to experiences of lack of energy, feeling tired and worn out, which are embraced in the ‘vitality’ concept. Fatigue/feeling tired and lack of energy have emerged as the most commonly reported symptoms in CKD 4–5 [50]. Moreover, it is well known that patients with advanced CKD have reduced physical functioning and performance [51,52] and that this is linked to experiencing fatigue [10] and that inactivity has an impact on fatigue in hemodialysis patients [53]. Our results confirm previous findings that GFR around 45 ml/min/1.73 m² seem to be a dividing line for drop in HRQoL, especially in PCS [19]. It also supports the hypothesis of a turning point in PCS at a relatively early and asymptomatic stage. This is further confirmed by others who have found physical fitness and functioning to be reduced already in earlier CKD stages to approximately 70% of the expected norm [51]. The loss of physical fitness and function demonstrated in this and other studies is alarming and has to be addressed already at a mildly diminished renal function. Enhanced physical activity and exercise training programmes to patients with renal insufficiency have been highlighted as interventions improving HRQoL [12,51]. Furthermore, resistance training has been proposed as a beneficial strategy also from an inflammation perspective [45,54]. Other interventions to enhance HRQoL in CKD patients, such as therapy optimization, management of anemia, sleep disturbances, depression, stress and anxiety and support in cognitive dysfunction have been suggested [12] as well as a comprehensive approach to a more patient-centered care [13]. From a nursing perspective, retaining or increasing HRQoL and well-being is a care goal [2] together with supporting and empowering patients to achieve more of health literacy, capability and autonomy. Interventions aiming at giving feedback and discussing the HRQoL outcomes face-to-face with the individual patient have been studied within oncology and diabetes care [55,56], and has also been suggested as a future pathway within renal care [57]. Especially in earlier stages of the disease these discussions might make the patient aware of possible growing decreases in function and well-being and may help the patient to timely find healthy coping strategies. Systematic implementation of monitoring and discussing HRQoL would not only facilitate the communication and improve the understanding of the patients’ perspective in a more holistic care approach, but also provide a tool to screen for and prioritize problems. This could then compose a base for supporting and improving the patients’ self-efficacy [56] and psychosocial well-being [55].

### Implications for practice

Systematic assessments of HRQoL already from earlier CKD stages could be a useful tool in renal care in order to explore and improve perceived health and well-being. The most affected HRQoL dimensions – perceptions of general health and physical health components - insist that recognition and management of them should be attended. The impairments in mental health components in CKD 5 stress the importance of psychosocial support to patients about to commence dialysis treatment. Also co-existing conditions and predictors like inflammation, CVD and also diabetes should be attended when assessing HRQoL in CKD patients.

### Implications for future research

These data suggest that HRQoL in CKD is not only related to the renal function level, but also to other conditions relating to CKD, like inflammation, CVD and diabetes. However, longitudinal studies are needed to confirm these findings. Follow-up studies on HRQoL from earlier CKD stages with a comprehensive approach are required, with a special interest in exploring alterations within CKD stage 3. Little is still known regarding illness perceptions and health experiences in this patient group, why more research is necessary in this field. There is also a need to further evaluate effective interventional strategies to enhance HRQoL in CKD patients, including secondary prevention of risk factors and co-existing conditions, educational and psychosocial support and programmes for improved physical activity.

## Conclusion

Having CKD implies impaired HRQoL, also in earlier stages of the disease. At the time for dialysis initiation HRQoL is substantially deteriorated. Co-existing conditions, such as inflammation and cardiovascular disease seem to be powerful predictors of impaired HRQoL in patients with CKD. Within routine renal care, strategies to improve function and well-being considering the management of co-existing conditions like inflammation and CVD need to be developed.

## Competing interests

The authors declare that they have no competing interests.

## Authors’ contributions

AP contributed for drafting of study design, data collection, analysis and interpretation of data, drafting of manuscript. BK, co-supervisor, contributed for study design and critical revision of manuscript for important intellectual content. CM contributed for acquisition of data and critical revision of manuscript for important intellectual content. BH, co-supervisor, contributed for study design and critical revision of manuscript for important intellectual content. SH, main supervisor, contributed for conception and study design, analysis and interpretation of data, statistical expertise, drafting and critical revision of manuscript for important intellectual content. All authors have read and approved the final manuscript.

## Supplementary Material

Additional file 1 **Table S1.** Participant flow chart. Click here for file
