# Supplementary material for: Health-related quality of life in different stages of chronic kidney disease and at initiation of dialysis treatment
Source: Health Qual Life Outcomes. 2012 Jun 18;10:71. doi: 10.1186/1477-7525-10-71 (PMC3511211; doi:10.1186/1477-7525-10-71)
Supplement: Additional file 1 — Table S1. Participant flow chart. [file 1477-7525-10-71-S1.doc]

*Additional file 1: Table S1*. Participant flow chart

| **Study**  **cohort, n** | **CKD**  **stage** | **Patients**  **in HRQoL**  **survey, n** | **Drop-**  **out***  **n** | **n** |  | **Participants, GFR ranges and**  **CKD stages, n** |
| --- | --- | --- | --- | --- | --- | --- |
| PROGRESS:  104 | →2 - 5 | →104 | 2 | →102 |  | *CKD stage 2* GFR range 60-69 ml/min/1.73m² 31 |
| SRR:  468 | →3 – 5 | →116 |  | →116 | 535 | *CKD stage 3*  GFR range 31-58 ml/min/1.73m² 23 |
| PAUS:  532 | →4 - 5 | →330 | 13 | →317 |  | *CKD stage 4*  GFR range 15-29 ml/min/1.73m² 87 |
|  |  |  | |  |  | *CKD stage 5*  GFR range 2-14 ml/min/1.73m² 394 |
|  |  |  | |  |  | *Controls*# 55 |
|  |  |  | |  |  | All participants 590 |

#Gfr≥80 ml/min, matched to patients in CKD stages 2-3. *drop-out due to incomplete responses
